# Innovative applications of artificial intelligence in zoonotic disease management

**DOI:** 10.1016/j.soh.2023.100045

**Published:** 2023-11-03

**Authors:** Wenqiang Guo, Chenrui Lv, Meng Guo, Qiwei Zhao, Xinyi Yin, Li Zhang

**Affiliations:** a Department of Animal Nutrition and Feed Science, College of Animal Science and Technology, Huazhong Agricultural University, Wuhan 430070, China; b College of Veterinary Medicine, Henan Agricultural University, Zhengzhou 450046, China

**Keywords:** Zoonotic diseases, Artificial intelligence, Epidemiological surveillance, Disease prediction, Early diagnosis, Drug development

## Abstract

Zoonotic diseases, transmitted between humans and animals, pose a substantial threat to global public health. In recent years, artificial intelligence (AI) has emerged as a transformative tool in the fight against diseases. This comprehensive review discusses the innovative applications of AI in the management of zoonotic diseases, including disease prediction, early diagnosis, drug development, and future prospects. AI-driven predictive models leverage extensive datasets to predict disease outbreaks and transmission patterns, thereby facilitating proactive public health responses. Early diagnosis benefits from AI-powered diagnostic tools that expedite pathogen identification and containment. Furthermore, AI technologies have accelerated drug discovery by identifying potential drug targets and optimizing candidate drugs. This review addresses these advancements, while also examining the promising future of AI in zoonotic disease control. We emphasize the pivotal role of AI in revolutionizing our approach to managing zoonotic diseases and highlight its potential to safeguard the health of both humans and animals on a global scale.

## Highlights

• AI enables accurate prediction of zoonotic disease outbreak and transmission pattern.
• AI-powered diagnostic tools expedite pathogen identification and containment.
• Virtual screening driven by AI algorithms accelerates discovery of new therapeutics.
• Responsible deployment of AI for zoonotic diseases.

## Abbreviations

AI: Artificial intelligence
ANN: Artificial neural networks
ARIMA: Autoregressive integrated moving average
CDRA: Call data record analysis
DL: Deep learning
DNN: Deep neural network
EBOV: Ebola virus
EDA: Exploratory data analysis
EVD: Ebola virus disease
FDA: Flexible discriminant analysis
GCN: Graph convolutional neural networks
GLM: Generalized linear models
HMD: Human monkeypox detection
IoMT: Internet of Medical Things
IoT: Internet-of-Things
LSTM: Long short-term memory
MAPE: Mean absolute percentage error
MARV: Marburg virus
MD: Molecular dynamics
ML: Machine learning
MLP: Multilayer perceptron
NiV: Nipah virus
RBM: Restricted Boltzmann machine
RNN: Recurrent neural network
RVF: Rift Valley fever
WNB: Weighted naive Bayes

## 1 Introduction

Zoonotic diseases are infectious diseases that can be transmitted between animals and humans and pose a significant threat to global public health [1]. The emergence and re-emergence of zoonotic diseases, such as coronavirus disease 2019 (COVID-19), Ebola virus disease (EVD), and monkeypox, highlight the need for innovative approaches to enhance disease prevention, early diagnosis, and effective treatment [2,3]. In recent years, artificial intelligence (AI) has emerged as a powerful tool in the field of healthcare and has shown great potential in addressing these challenges [[4], [5], [6]].

The integration of AI techniques with conventional disease control strategies offers novel prospects for understanding, predicting, and mitigating the impact of zoonotic diseases [7]. By leveraging advanced algorithms and machine learning (ML) models, AI can analyze vast amounts of complex data from diverse sources, ranging from environmental factors to genetic sequences, enabling researchers and public health authorities to make more informed decisions and implement proactive measures [8,9].

**Fig. 1:**
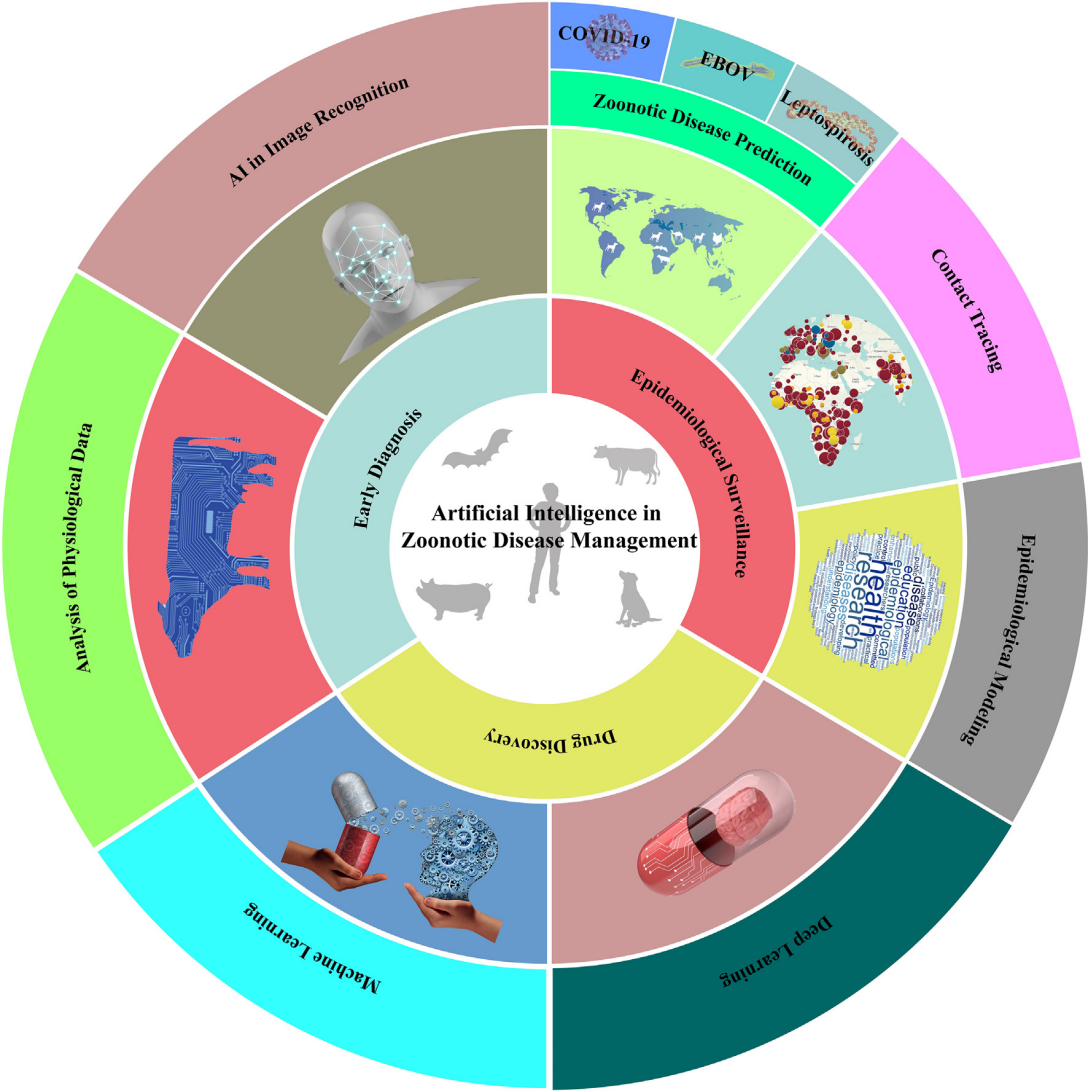
Overview of the various applications of artificial intelligence (AI) technology in zoonotic diseases.

In this review, we aimed to explore the innovative applications of AI in zoonotic diseases, with a specific focus on epidemiological surveillance, early diagnosis, and drug development (Table 1 and Fig. 1). We examined the current state of research in each of these domains, highlighting key advancements and discussing their implications for public health. Additionally, we identified the challenges and limitations of AI in zoonotic disease research, as well as opportunities for future development.

**Table 1:** Overview of the applications of AI in the management of zoonotic diseases.

Scope of application	Method/Purpose		Summary	Reference
Epidemiological surveillance	Disease prediction	COVID-19	AI-based RNN to accurately predict the prevalence profile of COVID-19.	[10]
			MLP neural network to predict cumulative incidence of COVID-19 in US counties.	[11]
		EVD	BioTEMS, an environmental modeling system for determining cryptic reservoir species of Ebola virus, the duration and location of latent reservoirs between outbreaks, and environmental epidemiological surveillance.	[12]
			Data analysis framework combining ANN and genetic algorithms for predicting EVD outbreaks from big data.	[13]
		Leptospirosis	Combination of data mining and ANN modeling to analyze, capture, and predict the occurrence of leptospirosis.	[14]
			Prediction of leptospirosis with potential for spatial analysis by combining neural network prediction models and principal component analysis.	[15]
		Other diseases	Monkeypox prediction technique combining multiple machine learning strategies (linear regression, decision trees, random forests, elastic nets and ARIMA, etc.).	[16]
			Prediction of future outbreaks of anthrax in livestock in Karnataka by machine learning techniques (GLM, generalized additive models, multiple adaptive regression splines and FDA).	[17]
			Epidemiological modeling analyzing the intrinsic links between diseases and ecological and social factors, data-driven by neural network techniques for RVFs.	[18]
			EBP-ANN algorithm to predict and mitigate the adverse effects of viral zoonoses on human health.	[19]
	Contact tracing		An intelligent application enables real-time contact tracing by capturing close proximity events between two smartphones running the application.	[20]
			COVICT employs real-time personal symptom data and contact tracing, utilizing IoT and gradient boosting algorithms.	[21]
			This model quantifies the number of infections, contacts, and duration of the epidemic between the appearance of primary cases and the detection of secondary cases.	[22]
			The modle utilized Call Data Record Analysis to extract location information from bedding, enabling the tracking of patients infected with the dengue virus and controlling the spread of the epidemic.	[23]
	Epidemiological modeling		A predictive modeling framework enhanced by AI is capable of forecasting the expected numbers of COVID-19 confirmed deaths, cases, and hospitalizations.	[24]
			The model can evaluate the temporal relationship between meteorological variables, entomological monitoring indices, and confirmed dengue fever cases.	[25]
			A novel machine learning-based framework that utilizes static and dynamic features of places to estimate the parameters of any epidemiological model, such as contact rate and recovery rate	[26]
Early diagnosis	Physiological data analysis		Predictive machine learning model combines patient signals, clinicopathologic data, and traditional early diagnostic results for early diagnosis of leptospirosis.	[27]
			The combination of RBM and hybrid ensemble learning method developed a prognostic model for early diagnosis of Nipah virus.	[28]
			Generalized Deep Convolutional Fuzzy Network based on Shuffle Shepherd Optimization approach for early diagnosis of COVID-19.	[29]
			Early diagnosis model of monkeypox virus based on MDiNFIS distinguishes monkeypox virus from other pox diseases.	[30]
			Early detection of monkeypox patients by weighted naïve Bayes, K-Nearest Neighbors and deep learning algorithms are realized.	[31]
	Image recognition		Model optimization strategies for early diagnosis of specific zoonotic diseases with AI algorithms and deep learning architectures.	[32,33]
			Smart drones with thermal imaging recognition assist in the early diagnosis of COVID-19.	[34]
			Combining Python and machine learning techniques (random forests, logistic regression, etc.) to analyze chest X-ray images for early diagnosis of COVID-19.	[35]
Drug development	Machine learning		Drug development for SARS-CoV-2 protein drug targets via enhanced sampling MD and ensemble docking in a supercomputer-driven pipeline.	[36]
			New deep neural network-based machine learning algorithm SSnet for COVID-19 drug screening.	[37]
			Gradient boosting tree ensemble-based supervised machine learning model and relies on in vitro data encoded as chemical fingerprints to identify specific molecular substructures of different COVID-19 potential drugs.	[38]
			Analysis of factors contributing to the development of drug resistance in Salmonella, *Listeria monocytogenes* and Campylobacter by machine learning methods (RrandomForest and XGBoost) and deep learning methods (multilayer perceptron, generative adversarial networks and autoencoders).	[39]
	Deep learning		Implementation of generative models optimized by transfer learning and reinforcement learning for the development of small molecule drugs for 3CL protease inhibition.	[40]
			A deep learning model specifically designed to predict the inhibitory activity of unknown compounds against MARV.	[41]
			Deep-AVPpred, the deep learning classifier, for predicting AVPs in protein sequences.	[42]
			Conceptual DL framework with the function of identifying, analyzing and predicting the performance of drugs at different stages.	[43]
			Predicting cross-species biomarkers based on viral mRNA or protein sequences with deep learning models advances influenza drug development.	[44]

By highlighting the potential of AI in zoonotic disease control, we aim to contribute to the growing body of knowledge in this field and stimulate further research. Ultimately, harnessing the power of AI to combat zoonotic diseases holds promise for improving global health outcomes, reducing morbidity and mortality, and preventing future pandemics.

## 2 Application of AI in epidemiological surveillance

### 2.1 AI advancements in predicting zoonotic diseases

AI technology has been increasingly utilized for the prediction and management of zoonotic diseases, including COVID-19, EVD, leptospirosis, and various other diseases [10,45]. AI-based algorithms analyze vast datasets of human and animal health information, environmental factors, and pathogen characteristics to predict disease outbreaks and transmission patterns [[46], [47], [48]].

#### 2.1.1 COVID-19

**Fig. 2:**
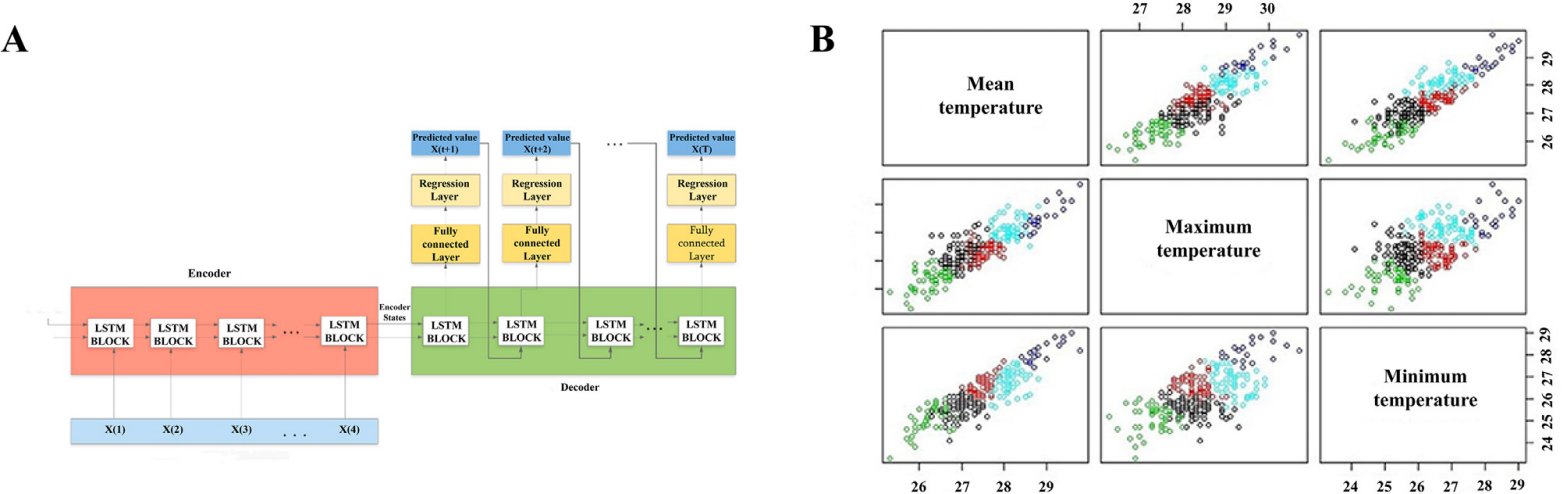
AI advancements in predicting zoonotic diseases. **(A)** Intelligent neural network for predicting the COVID-19 pandemic [10]. **(B)** Clustering analysis of temperature data using machine learning (ML) techniques, with five colors representing five distinct clusters for the analysis and prediction of the occurrence of Hookworm Disease [14]. **(C)** An ML model employing techniques such as linear regression, decision trees, random forests, elastic nets, and for the prediction of monkeypox [16].

AI algorithms can efficiently process and analyze extensive datasets derived from diverse sources, including healthcare records, social media, and mobility patterns [49]. By identifying correlations and patterns within these data, AI can offer valuable insights into the spread of the virus, encompassing infection rates, transmission dynamics, and hotspot identification [50]. Moreover, AI-powered predictive models leverage historical data and real-time information to forecast pandemic trends [51]. One study used a dataset composed of publicly available data from the World Health Organization and Johns Hopkins University [10]. They employed a recurrent neural network (RNN) to accurately predict the epidemic curve of COVID-19, specifically focusing on new daily cases (Fig. 2A). To assess the efficacy of the model, the study compared its predictions against observed data using metrics such as the root mean square logarithmic error, root mean square error, and mean absolute error (MAPE). The results demonstrate the accuracy of the model in predicting the progression of COVID-19, highlighting the utility of AI-based models in epidemic prediction.

Another study analyzed the epidemic curve of the COVID-19 pandemic by constructing a disease prediction model using AI RNNs [52]. The researchers curated the training dataset using publicly available data from the World Health Organization and Johns Hopkins University. They employed RNNs with gated recurrent units and long short-term memory (LSTM) to build two prediction models. The outcomes of these predictions revealed recurring peaks in the epidemic curve with minimal discrepancies between the predicted and validated data and trends. In addition, the authors proposed future infection peaks based on the model, which aligned with the actual situation, further substantiating the reliability of the model.

In a separate study, a multilayer perceptron (MLP) neural network was employed to predict the cumulative incidence of COVID-19 across the United States [11], wherein a comprehensive database of 57 explanatory variables was compiled. The findings demonstrated that the MLP neural network accurately projected the cumulative incidence rate, exhibiting a correlation of 65% with the actual situation. Furthermore, the authors identified significant associations between the precise prediction of COVID-19 incidence rates and factors, such as ischemic heart disease, pancreatic cancer, socioeconomic status, and environmental factors. These findings not only hold significance for predicting COVID-19 incidence rates but also aid public health decision-makers in comprehending the impact of potential risk factors associated with county-level COVID-19 incidence rates.

#### 2.1.2 EVD

AI technology has demonstrated its invaluable role in predicting potential hosts for EBOV, aiding researchers and public health authorities in understanding and mitigating the risk of EBOV transmission from wildlife to humans. This proactive approach played a crucial role in preventing Ebola outbreaks [53]. Kollars Jr et al. proposed a method that involves identifying the hidden reservoir species of the EBOV, analyzing the duration and locations of latent reservoirs during intermittent periods of EBOV outbreaks, and conducting environmental epidemiological monitoring [12]. They developed the environmental modeling system BioTEMS to analyze mammals, arthropods, plants, and protozoa in Sierra Leone, with the aim of determining the species most likely to serve as hidden storage hosts for EBOV and their distribution. The results indicated that insects of the order Diptera, specifically those in the genus *Chrysops*, were potential secondary reservoir hosts and mechanical vectors, providing insights for reducing EBOV transmission.

With the increasing use of big data, researchers have focused on disease prediction strategies that utilize this vast amount of information [10]. In one study, a data analysis framework was designed to predict EVD outbreaks using big data from epidemiological studies [13]. The framework proposes a hybrid algorithm that combines ML algorithms, specifically artificial neural networks (ANNs) and genetic algorithms, implemented on the Apache Spark and Kafka frameworks. This approach aims to extract new information from large data repositories in the fields of environment, epidemiology, and immunology. Using Nigeria as a case study, this study successfully predicted the scale, timing, and duration of future EVD outbreaks, thus offering an effective means for EVD disease prediction.

In most Ebola infection cases, patients succumb to the disease before their bodies generate antibodies [54]. This highlights the need for improved EVD prediction techniques to achieve greater accuracy. In a separate study, researchers developed a hybrid neural network by combining various data-mining techniques and hybrid models to enhance the intelligence and accuracy of EVD prediction [54]. The techniques employed include logistic regression, random forest, and hybrid neural networks. The results demonstrated that employing the random forest classification strategy could improve the accuracy of traditional Ebola predictions by up to 100%, thereby providing the potential for analyzing and enhancing the accuracy of EVD prediction systems.

#### 2.1.3 Leptospirosis

Leptospirosis, a zoonotic disease influenced by weather and environmental changes, can be detected early through the analysis of diverse data sources using AI algorithms [55]. These sources include weather patterns, environmental factors, and animal populations. Rahmat et al. conducted a study that analyzed, captured, and predicted the occurrence of leptospirosis by combining data mining and ML techniques (Fig. 2B) [14]. Their specific focus was on the relationships between the disease and temperature, rainfall, and relative humidity. The study began with exploratory data analysis (EDA) using graphical methods to ascertain the optimal time lag for rainfall analysis. In contrast, non-graphical methods were employed for temperature analysis. Subsequently, an ANN model was developed to enhance the overall accuracy, sensitivity, and specificity of the disease prediction strategies. They collected data from the Seremban district in Malaysia for their predictions and compared the results with those of traditional forecasting models [14]. The findings demonstrated that the ANN model achieved the highest accuracy, sensitivity, and specificity, with values of 84.00%, 86.44%, and 79.33%, respectively. Furthermore, the EDA method improved the accuracy of the prediction model by 13.30–31.26% compared to the baseline model.

In another study focusing on leptospirosis, a neural network prediction model was used for disease forecasting [15]. This study also incorporated spatial analysis using principal component analysis to explore the potential for enhancing the prediction model by incorporating soil and land-use components. Comparisons between traditional prediction models and the strategy proposed in this study revealed a remarkable improvement of 31.26% in accuracy and 35.43% in sensitivity for the prediction model compared with conventional non-lagged prediction models. The authors also identified factors, such as soil clay content and residential land use, as important determinants of leptospirosis. These findings provide new insights and considerations for disease prediction in leptospirosis.

#### 2.1.4 Other zoonotic diseases

AI plays a significant role in the prediction of various zoonotic diseases. Monkeypox is a zoonotic disease characterized by fever, rash, and swollen lymph nodes, with potential health consequences including skin lesions and scarring [48]. Recently, this has become a prominent topic in zoonotic disease research. One study proposed a prediction method for monkeypox using ML techniques [16]. This study employed multiple techniques, such as linear regression, decision trees, random forests, elastic nets, and Auto Regressive Integrated Moving Average (ARIMA), achieving a high prediction accuracy of 0.9267 R^2^ for monkeypox. R^2^, also known as the coefficient of determination, represents the statistical measure of how well the regression model fits the observed data. It indicates the proportion of the variance in the dependent variable that can be accounted for by the independent variable in the model. An R^2^ value ranges from 0 to 1, with values closer to 1 indicating a better fit between the model and data. In this study, the R^2^ value of 0.9267 suggests that approximately 92.67% of the variation in monkeypox can be explained by the multiple regression models used. The high prediction accuracy indicates that the models are reliable tools that could help researchers identify effective interventions for reducing the impact of this disease. Therefore, this prediction model serves as a valuable tool for ongoing studies of monkeypox [16].

Anthrax is an acute infectious disease affecting humans and animals, caused by the bacterium *Bacillus anthracis* [56]. Early detection of anthrax outbreaks is crucial for minimizing the number of cases, deaths, and the risk of disease spread. A recent study aimed to develop a disease prediction model using ML techniques to forecast anthrax outbreaks in livestock in Karnataka [17]. The goal was to achieve early detection of anthrax cases. The authors employed an ML model developed with version 3.1.3 of the R statistical software. They combined various data mining regression and classification models, including generalized linear models (GLMs), generalized additive models, multiple adaptive regression splines, and flexible discriminant analysis (FDA). Anthrax occurrence data from the Animal Husbandry Department in Bangalore, Karnataka, India, were utilized. The study successfully identified susceptible areas where the next anthrax outbreak is likely to occur. Thus, this disease prediction model could serve as an early warning system for anthrax outbreaks in Karnataka livestock.

Rift Valley fever (RVF) is a zoonotic viral disease that can cause mass die-offs in livestock and has a high fatality rate in affected human populations [57]. Despite its significant impact, little is known about the occurrence of RVF and factors influencing its transmission. One study aimed to conduct data-driven epidemiological modeling of RVF using neural network techniques [18]. By integrating landscape archaeology, historical evidence, climate data, and human behavioral evidence collected through ethnoarchaeological research, the study proposes a human-animal paleopathology application framework. This framework analyses the inherent connections between diseases and ecological and social factors, aiding in addressing the threat of zoonotic diseases resulting from climate warming.

Viral zoonotic diseases pose serious threats to human and animal health [58]. Choubey et al. used an enhanced backpropagation ANN (EBP-ANN) algorithm to predict and mitigate the adverse impacts of viral zoonotic diseases on human health [19]. Viral datasets were collected and preprocessed using Z-score normalization. Subsequently, they extracted viral data features using a dynamic angle projection pattern and employed GAs to select more accurate feature data. The evaluation of the performance of the system revealed superior prediction accuracy compared to existing techniques, establishing its efficacy in improving viral disease prediction efficiency.

In summary, AI technology has been applied in various ways to predict and mitigate zoonotic diseases including COVID-19, EBOV, and leptospirosis. The application of AI in zoonotic disease prediction not only enhances our ability to safeguard both human and animal health but also contributes to a more proactive and effective approach to managing potential pandemics.

### 2.2 AI for contact tracing

Contact tracing plays a vital role in managing infectious disease outbreaks and is considered a crucial public health measure [59]. The purpose of contact tracing is to identify and monitor individuals who may have been in contact with an infected person, thereby preventing further transmission [60]. Manual contact tracing is labor-intensive; however, the use of AI and big data analytics can enhance and expedite this process.

In the case of the COVID-19 pandemic, a primary challenge arises from the potential for infected individuals to remain asymptomatic or pre-symptomatic while retaining the capacity to transmit the virus [61]. Consequently, it is essential to identify and track contacts of known cases to minimize the spread of the virus. Throughout the pandemic, various digital contact tracing solutions have employed location data from mobile devices and ML algorithms to identify potential contacts [[62], [63], [64]]. Ferretti et al. proposed an application based on the existing technology that facilitates real-time contact tracing [20]. This application establishes a log of close contacts and captures close proximity events between two smartphones running the application. In the event that an individual is diagnosed with COVID-19, the application promptly sends automated and anonymous risk notifications to those who have close contacts, accompanied by a request for self-isolation. Consequently, if a sufficiently high proportion of the population utilizes contact tracing mobile applications, it would be adequate to halt the spread of the epidemic [20]. This AI-based contact tracing system enabled the rapid issuance of contact alerts and recommendations for isolation, effectively mitigating the spread of the pandemic. Another notable system proposed by Wahid et al. is an Internet-of-Things (IoT)-based COVID-19 detection and monitoring system. COVICT employs real-time personal symptom data and contact tracing, utilizing device-to-device (D2D) communication and gradient-boosting algorithms to determine proximity and contact duration [21]. This system achieved impressive accuracy, with a classification error of 2.9%, sensitivity of 96.5%, and specificity of 97.7% [21]. Keeling et al. developed a predictive model to assess the effectiveness of contact-tracing strategies in the UK. By combining detailed survey data on social contacts from over 5800 respondents with a predictive model of contact tracing and control, they evaluated the potential impact of contact tracing and identified missed secondary cases before severe symptoms emerged. The study demonstrated the effectiveness of the UK's contact tracing strategy in identifying a significant proportion of infections [21].

To control the spread of monkeypox in nonendemic countries, Ko et al. developed a stochastic model based on Gillespie's random kinetic algorithm [22]. This model quantifies the number of infections, contacts, and duration of the epidemic between the appearance of primary cases and detection of secondary cases. The model considers delays in tracking contacts and establishes different scenarios to address various situations. The findings of this model highlight the significant impact of self-reporting behavior exhibited by primary cases on both the scale and duration of the outbreak. Compared to non-self-reporting primary cases, those who self-reported had an 86 % reduction in the number of infections and contacts [22]. This research not only provides a scenario simulation for contact tracing but also emphasizes the importance of the immediate detection of primary cases.

Digital contact-tracing solutions have been implemented in various locations, typically relying on mobile applications that utilize Bluetooth technology to identify close contacts of an infected individual [65,66]. When an individual is diagnosed with COVID-19, they can input this information into the application, which records interactions with other application users who have had contact with the infected person using Bluetooth technology [67]. These users then receive notifications that alert them to potential infection risks and provide guidance on adopting the required precautions, such as self-isolation or testing for COVID-19.

The effectiveness of these AI-based contact-tracing solutions depends on their widespread adoption and usage. If a substantial proportion of the population utilizes these applications to accurately report their infection status, the spread of the virus can be effectively curtailed. However, these approaches have challenges and limitations. Not everyone possesses a smartphone or is willing to use such applications, thereby limiting their coverage [68]. Furthermore, concerns regarding privacy and data security emerge as a consequence of the collection and processing of personal user data [69]. In summary, digital contact-tracing solutions can serve as valuable tools for mitigating the spread of zoonotic diseases, including COVID-19. However, their effectiveness relies on widespread adoption and usage while carefully considering privacy and data security aspects.

### 2.3 AI advancements in epidemiological modeling

In addition to contact tracing, AI plays a crucial role in epidemiological modeling, enabling the prediction of disease spread in populations over the long term. By utilizing advanced ML algorithms, these models integrate disease parameters, human behavior, and environmental factors to generate highly accurate predictions [47,70]. Throughout the COVID-19 pandemic, researchers have employed various modeling techniques, including computational agent-based, longitudinal prediction, and meta-population models combined with mobility data [71].

Arik et al. proposed a predictive modeling framework enhanced by AI capable of forecasting the expected numbers of COVID-19 confirmed deaths, cases, and hospitalizations on a nationwide scale for both the United States and Japan over the upcoming 4-week period [24]. During the prospective deployment, MAPE for predicting COVID-19-related deaths remained below 8% (United States) and 29% (Japan), whereas the cumulative MAPE remained below 2% (United States) and 10% (Japan). This framework enabled counterfactual simulations, demonstrating the crucial role of sustained non-pharmaceutical interventions and vaccination in achieving faster recovery from the pandemic. Delayed intervention measures can have harmful effects, and different vaccination strategies can be studied using this framework [24].

Currently, there is a dearth of successfully developed epidemiological models for monitoring dengue fever in nonendemic areas [25]. A study by Chang et al. aimed to identify an optimal model for dengue fever surveillance in non-endemic regions by evaluating the temporal relationships between meteorological variables, entomological monitoring indices, and confirmed dengue fever cases. The analysis was based on epidemiological, entomological, and meteorological data collected between 2005 and 2012 from Kaohsiung City, Taiwan, China. The model incorporates mosquito larval indices, namely the Breteau index, container index, and house index, along with an adult index, to analyze dengue fever data. Poisson regression was employed to select the best subset of variables and time delays for predicting the number of dengue fever cases. The final result of the multivariate analysis was determined based on the minimum Akaike information criterion value [25]. Subsequently, a multivariate logistic regression model was applied to the selected indices and variables to assess the accuracy of predicting dengue fever. The accuracy rates of the model for predicting dengue fever cases using the one adult, Breteau, Container, and House indices were 83.8%, 87.8%, 88.3%, and 88.4%, respectively. The predictive thresholds for the individual models (one adult, Breteau, container, and house indices) were 0.97, 1.16, 1.79, and 0.997, respectively.

Furthermore, Gatta et al. proposed a novel machine learning-based framework that utilizes static and dynamic features of places to estimate the parameters of any epidemiological model, such as contact rate and recovery rate [26]. By employing graph convolutional neural networks (GCNs) and LSTMs, they investigated the spatiotemporal characteristics of mobile data to infer the parameters of the susceptible–infected–recovered and the susceptible-infected-recovered-dead models.

These AI-driven epidemiological models have played a significant role in predicting infection curves, assessing the impact of public health interventions, and providing information for policy decision-making. As efforts toward pandemic prevention and control continue, AI-based modeling will serve as an essential tool for decision-makers to mitigate the impact of future outbreaks [72].

## 3 Enhancing early diagnosis of zoonotic diseases through AI advancements

Advancements in AI have contributed greatly to the early diagnosis of zoonotic diseases, benefiting both human and animal health. Analysis of physiological data using AI algorithms enables the detection of subtle changes in vital signs or biomarkers, aiding in the identification of potential infections or disease outbreaks. Additionally, the application of AI to image recognition allows for the accurate and rapid identification of zoonotic disease indicators in medical images, facilitating early intervention and containment measures. These advances have played crucial roles in improving disease surveillance, prevention, and control strategies, ultimately mitigating the risks associated with zoonotic diseases.

### 3.1 Analysis of physiological data

The use of AI in the analysis of physiological data holds substantial significance for the timely diagnosis of zoonotic diseases, which can affect both human and animal populations [31]. By applying AI algorithms to analyze complex physiological data, researchers can identify patterns and markers that may indicate the presence of a zoonotic disease, facilitating timely intervention and prevention measures [6].

**Fig. 3:**
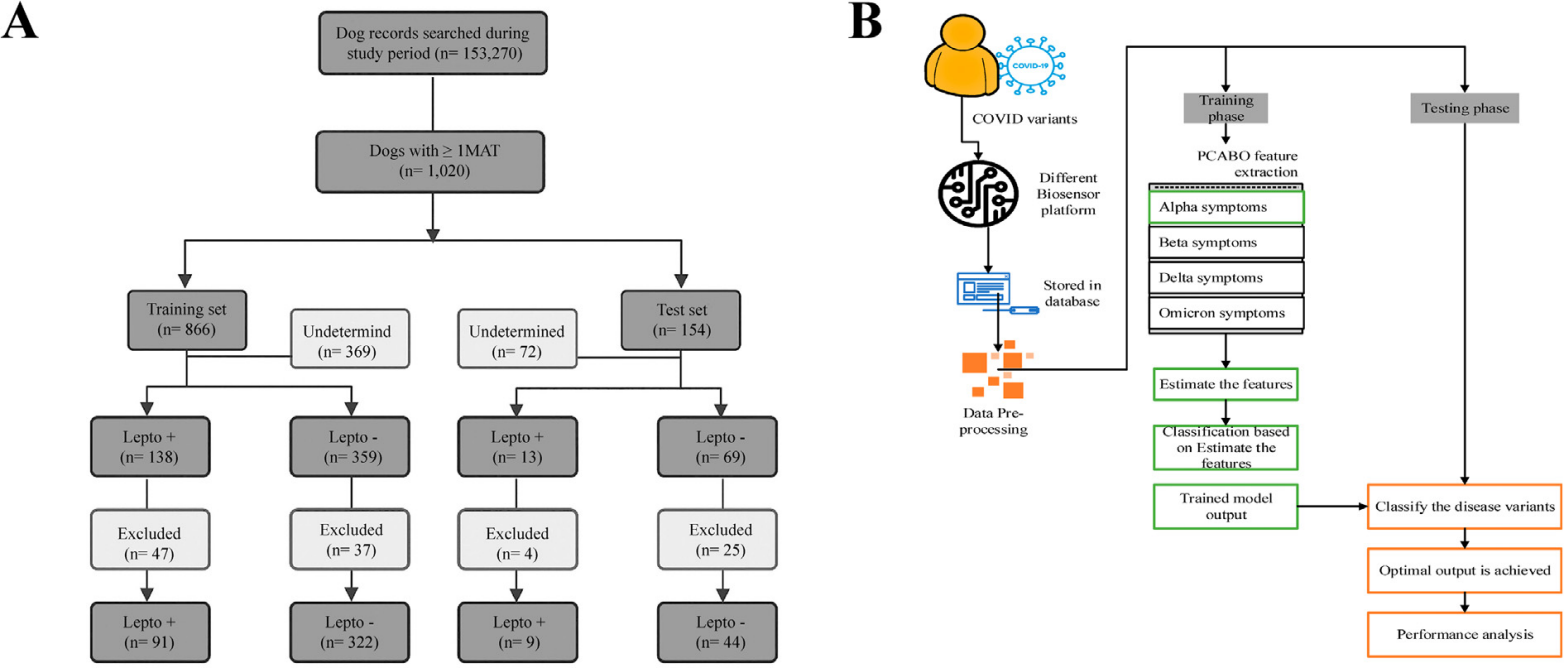
Enhancing early diagnosis of zoonotic diseases through AI advancements. **(A)** Employing Restricted Boltzmann Machine (RBM) methodology for processing clinical features to facilitate early diagnosis of NiV [27]. **(B)** Data collection from sensors, feature extraction using the African buffalo optimization method, and subsequent analysis and assessment of features by Shuffle Shepherd Optimization-based Generalized Deep Convolutional Fuzzy Network (SSO-GDCFN) for the diagnosis of COVID-19 [29].

ML, a subset of AI, plays a critical role in the analysis of physiological data [73]. Traditional methods for early diagnosis of leptospirosis in animals have limitations in terms of sensitivity and diagnostic time [27]. However, one study was conducted using a predictive ML model that combines patient signals, clinicopathological data, and traditional early diagnosis results [28]. The researchers collected data from both diseased and healthy dogs to train the model, resulting in 100% sensitivity in early diagnostic strategies for leptospirosis (Fig. 3A). Kannan et al. developed a prognostic model for the early diagnosis of Nipah virus (NiV) using ML techniques [28]. They combined a range of clinical factors such as symptoms, disease incubation period data, and routine blood test results confirmed by laboratory technicians. The study proposes an approach called the restricted Boltzmann machine (RBM) to handle a large number of clinical features. Furthermore, a hybrid ensemble learning method was employed to determine NiV infection in patients after feature selection using RBM. The model achieved an accuracy of 88.3% after validation.

Additionally, AI-based analysis of physiological data enables the real-time monitoring and surveillance of zoonotic diseases [74]. By continuously analyzing data from wearable devices, environmental sensors, and animal health records, AI algorithms can promptly identify disease outbreaks or emerging zoonotic threats [75]. Alam et al. introduced a method called the shuffle shepherd optimization-based generalized deep convolutional fuzzy network (SSO-GDCFN) for the early diagnosis of COVID-19 (Fig. 3B) [29]. They collected data from various sensors, including potentiometers, blood pressure sensors, graphene field-effect-transistor (G-FET)-based sensors, and electrochemical sensors. After data preprocessing, we used the principal component of African buffalo optimization method for feature extraction. Subsequently, the features were analyzed and evaluated using SSO-GDCFN to determine whether they could be diagnosed as COVID-19. The results demonstrate an accuracy of 99.99% using this method.

Moreover, AI facilitates the development of early diagnosis models for zoonotic diseases [76]. Tom et al. proposed a neuro-fuzzy earl diagnosis model for the monkeypox virus, allowing differentiation from other pox diseases [30]. The diagnostic model comprises a knowledge base, neuro-fuzzy inference engine, and decision support engine. By inputting a patient's physiological data and conducting a preliminary diagnosis based on the database, the model ensures the accuracy and precision of the diagnostic results using the intelligent neuro-fuzzy inference system (MDiNFIS). Currently, the model is available as software for use by patients and medical institutions. Saleh et al. presented an AI technology model called the human monkeypox detection (HMD) strategy for the early diagnosis of monkeypox in patients [31]. The model comprised two main stages: selection and detection. The selection stage aims to identify the most relevant features corresponding to the disease, and the detection stage provides rapid and accurate detection based on the effective data obtained from the selection stage. The model comprises three diagnostic algorithms: weighted naïve Bayes, weighted K-nearest neighbors, and deep learning (DL) algorithms [31]. These algorithms are combined using a novel weighted voting method to obtain optimal diagnostic results. Experimental results show that, compared to other modern strategies, the HMD strategy achieves the highest accuracy, precision, and recall rates (98.48%, 91.1%, and 88.91%, respectively).

The AI-driven analysis of physiological data plays a crucial role in the early diagnosis of zoonotic diseases. By integrating multimodal data, utilizing ML algorithms, and enabling real-time monitoring, AI can empower researchers and healthcare professionals to detect and respond to zoonotic infections at their earliest stages. This proactive approach is vital to prevent outbreaks, safeguard human and animal populations, and improve overall public health.

### 3.2 Application of AI in image recognition

Indeed, the application of AI in image recognition has a significant impact on the early diagnosis of zoonotic diseases in humans and animals [77,78]. By analyzing and interpreting visual data, AI algorithms can accurately identify patterns, anomalies, and specific markers associated with such diseases [79,80]. This technology enables healthcare professionals and veterinarians to detect infections, monitor their spread, and implement prompt interventions to prevent outbreaks [81].

AI-based image recognition systems can quickly and efficiently process large volumes of medical and veterinary images [79]. They can detect subtle changes in tissues, organs, or cells, which may indicate the presence of a zoonotic disease [82]. Early detection allows for timely treatment and containment strategies, reducing the risk of transmission and minimizing its impact on public health [83]. Research studies have explored the use of AI algorithms and DL architectures, such as ResNet50, EfficientNetB3, EfficientNetB7, and EfficientNet-B0, for the early diagnosis of specific zoonotic diseases, such as monkeypox [32,33]. These studies have shown promising results, achieving high accuracy rates in detecting skin lesions associated with monkeypox. By collecting image data from around the world and using transfer learning techniques, researchers aim to enhance the accuracy of the early diagnosis of various zoonotic diseases.

Furthermore, AI-powered image recognition systems can overcome the limitations posed by human error or subjectivity in the interpretation of complex visual data [84]. For example, in the early diagnosis of COVID-19, unmanned aerial vehicles equipped with thermal imaging cameras can intelligently identify masks and detect abnormal body temperatures in crowds [34]. This technology enables the detection of potential COVID-19 patients without human intervention, thereby facilitating early diagnosis and data collection. Similar AI-assisted systems have been developed for the early detection of chicken diseases. Using smart mobile devices to capture fecal images, ensemble networks composed of fine-tuned convolutional neural networks have achieved impressive accuracy rates [85,86]. These advancements in AI technology offer hope for the early detection of various zoonotic diseases affecting poultry and other animals. Another study proposed the utilization of ML techniques, such as random forest, logistic regression, naïve Bayes, and support vector machines implemented in Python to classify a series of chest X-ray images, including in cases of viral pneumonia as well as COVID-19 and of uninfected individuals [35]. The study gathered over 1400 images from the Kaggle platform, and the results demonstrated that the model achieved accuracy, sensitivity, and specificity exceeding 90% for distinguishing between common influenza and COVID-19 [35,87]. This finding is significant for the diagnosis of COVID-19 and makes a valuable contribution to the fight against the disease [35]. Additionally, innovative devices combining reverse transcription loop-mediated isothermal amplification with AI algorithms have been proposed for the sensitive and specific identification of zoonotic diseases, such as COVID-19. These handheld smart devices demonstrate high analytical sensitivity and specificity, surpassing the current gold-standard diagnostic methods.

The integration of AI-based image recognition for the early diagnosis of zoonotic diseases is crucial. It provides healthcare professionals and veterinarians with a powerful tool for swiftly identifying and responding to potential outbreaks, thereby safeguarding human and animal health.

## 4 Discovery and development of drugs for zoonotic diseases

AI technologies, including ML and DL, have revolutionized the discovery and development of drugs for zoonotic diseases. Advanced AI techniques leverage large datasets and intricate data analyses to expedite drug discovery.

### 4.1 Machine learning

ML, a key component of AI, plays a significant role in the discovery and development of drugs for zoonotic diseases [88]. By employing algorithms that can learn from data, make predictions, and take decisions, ML enables researchers to analyze large datasets, identify patterns, and generate insights that facilitate the development of new drugs [43,89].

**Fig. 4:**
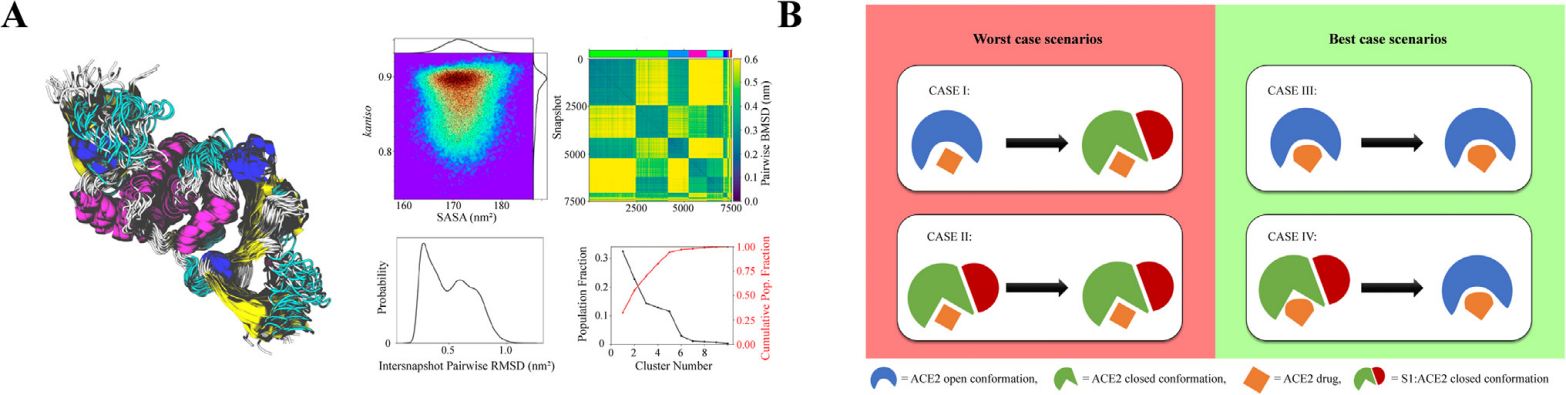
Discovery and development of drugs for zoonotic diseases. **(A)** Development of drugs through rapid exploration of the conformational space of protein drug targets to identify the most effective targeted drugs against SARS-CoV-2 [36]. **(B)** A machine learning (ML) algorithm, SSnet, based on deep neural networks, employed for the screening of potential drugs. The diagram depicts the proposed mechanism of action of putative drugs inhibiting the ACE2 (angiotensin-converting enzyme 2):S1 binding. Cases I and II represent the unexpected stability of the ACE2:S1 complex. Cases III and IV depict the stabilization of the open ACE2 conformation due to allosteric interference at the ACE2 binding interface by ACE2:S1 complex inhibitors [37].

A crucial application of ML in drug research is the identification of potential drug targets. ML algorithms can analyze biological and genetic data to identify molecules, proteins, or genes that participate in disease pathways [90]. This information helps researchers focus their efforts on developing drugs that interact with these targets, leading to more effective therapies [91]. Acharya et al. proposed a strategy for drug development by rapidly exploring the conformational space of the SARS-CoV-2 protein drug targets (Fig. 4A) [36]. This approach utilizes enhanced sampling molecular dynamics (MD) and ensemble docking in a supercomputer-driven pipeline for in silico drug discovery. It involves docking compound libraries against representative protein-binding site conformations, considering the dynamic nature of the binding sites, to identify the most effective targeted drugs against SARS-CoV-2. Researchers also aim to further optimize this strategy using methods such as quantum mechanics and ML.

ML also plays a vital role in virtual screening. By training models of known drug-target interactions, ML algorithms can predict the likelihood of a compound binding to a target of interest [92]. This approach enables researchers to prioritize and select the most promising drug candidates for further investigation, thereby saving time and resources [93]. In the face of unexpected crises such as COVID-19, utilizing AI methods to explore existing drugs as potential treatments for the disease has great potential. Karki et al. introduced a new ML algorithm, SSnet, based on deep neural networks (DNNs) for drug screening in the context of COVID-19 (Fig. 4B) [37]. They employed SSnet to screen a large compound library containing 750,000 compounds to rapidly identify potential drugs for urgent zoonotic disease cases similar to the approach employed for COVID-19 cases. They also made this strategy accessible through an open-access website, allowing researchers from different fields to use it. SSnet is a tool used to screen large molecular libraries to identify drugs with potential therapeutic effects. Another ML model for drug discovery and screening against COVID-19 focused on the molecular substructures of drugs [38]. This method utilizes a supervised ML model based on a collection of gradient-boosting trees and relies on in vitro data encoded as chemical fingerprints to identify specific molecular substructures of different potential drugs. The authors employed this model to screen for optimal repurposing drug candidates among drugs approved by the Food and Drug Administration.

Furthermore, ML contributes to the prediction of antimicrobial drug resistance, which is a crucial consideration for the development of drugs for zoonotic diseases [94]. An intelligent strategy has been developed to analyze factors contributing to multidrug resistance in poultry pathogens associated with zoonotic infections [39]. In the study, 1635 fecal and soil samples were collected from 42 poultry flocks and 11 farms in the southeastern United States. They employed two traditional ML methods (random forest and XGBoost) and three DL methods (MLP, generative adversarial networks, and autoencoders) to analyze significant factors influencing the development of drug resistance in *Salmonella*, *Listeria*, and *Campylobacter*. Based on the findings of this study, relevant recommendations are proposed to reduce the emergence of antimicrobial resistance.

ML plays a crucial role in the research and development of drugs for the treatment of zoonotic diseases. Its ability to analyze complex datasets and predict drug-target interactions accelerates drug discovery. By harnessing the power of ML, researchers can develop targeted therapies and advance the field of medicine.

### 4.2 Deep learning

DL algorithms in AI play crucial roles in drug discovery and development [95]. These algorithms can analyze vast amounts of biological and chemical data, enabling the identification of potential drug targets, prediction of drug properties, and acceleration of the discovery of new therapeutic compounds [96].

A significant application of DL in drug development is virtual screening. By training on large databases of molecules and their properties, DL models can accurately predict the likelihood of a specific molecule binding to a specific target [95]. This ability allows researchers to narrow down the search for potential drug candidates, saving valuable time and resources [97]. For example, one study developed small-molecule drugs that potentially inhibit the chymotrypsin-like (3CL) protease, a target protein of SARS-CoV-2, using a DNN generation and prediction model [40]. The generation model was optimized using transfer and reinforcement learning, focusing on the chemical space corresponding to the protease inhibitors. Multiple physicochemical property filters and virtual screening scores were used in the final screening process. Kumari et al. developed a DL model using resampling techniques specifically for the Marburg virus (MARV) [41]. This model was used in a virtual screening process to predict the inhibitory activities of unknown compounds against MARV. A resampling technique was used to address the issue of imbalanced data. The study achieved a high accuracy rate of 95% in screening for active lead molecules against MARV, utilizing various databases such as ChemDiv, ChEMBL antiviral library, plant chemical database, NCI divsetIV natural products, and the natural compound ZINC database.

DL algorithms also aid in de novo drug design, allowing the computational generation of new compounds [98]. By leveraging generative models, these algorithms can explore the chemical space and propose novel molecules with desired properties, enabling the discovery of unique drug candidates that traditional methods may have overlooked [99]. Sharma et al. proposed a DL classifier called Deep-AVPpred to predict antiviral peptides (AVPs) in protein sequences [42]. The classifier utilized transfer learning in DL algorithms and achieved an accuracy of 94%. In addition, deep-AVPpred has been used to discover novel AVPs in the human interferon-alpha family of proteins.

Furthermore, DL enhances the optimization of lead compounds by predicting their pharmacokinetic properties, such as absorption, distribution, metabolism, and excretion [100]. DL models assist in the selection of promising drug candidates for further development, streamlining the drug discovery pipeline, and increasing its efficiency [101]. Recently, a research study proposed a conceptual DL framework consisting of eight components, each responsible for identifying, analyzing, and predicting the performance of drugs at different stages [43]. This model can provide predictions and relevant recommendations for various stages of drug development with the aim of alleviating the time-consuming limitations of traditional drug development and experimental methods.

DL also facilitates the analysis of omics data, including genomics, proteomics, and metabolomics [102]. By integrating and interpreting these complex datasets, DL algorithms can identify biomarkers, understand disease mechanisms, and personalize treatment approaches [103]. The influenza A virus is the primary causative agent of high morbidity and mortality in zoonotic diseases. One study employed DL models to predict viral hosts based on viral mRNA or protein sequences to advance drug development for influenza by predicting cross-species biomarkers [44]. The model utilizes training data obtained from nucleotide sequences collected from the NCBI Influenza Virus and Influenza Research Databases. After classifying the data, various neural network interpretation methods were used to analyze the trained model and identify interesting candidate biomarkers for zoonotic disease transitions, which are of significant importance in drug development.

DL in AI has significant implications for drug research and development of zoonotic diseases. Its ability to analyze complex data, predict drug-target interactions, and accelerate the discovery of novel compounds has revolutionized the field. By harnessing the power of DL, researchers can expedite the identification of new treatments and ultimately improve healthcare outcomes for patients worldwide.

## 5 Discussion and perspective

In this review, we discuss innovative applications of artificial AI in zoonotic diseases, focusing on disease prediction, early diagnosis, and drug development. The integration of AI techniques with traditional disease control strategies has shown great potential for advancing our understanding of zoonotic diseases and improving public health outcomes.

One of the major contributions of AI to the study of zoonotic diseases is its ability to predict outbreaks and identify high-risk areas. By analyzing vast amounts of data from various sources, such as environmental factors, animal migration patterns, and human behavior, AI algorithms can generate accurate and timely predictions of disease emergence [104]. This will enable public health authorities to implement preventive measures and allocate resources effectively, ultimately reducing the impact of zoonotic diseases on human populations.

Furthermore, AI has revolutionized early disease diagnosis by improving the speed and accuracy of disease detection. AI can be used to analyze intricate patterns and biomarkers found in medical imaging, genetic data, and clinical records to identify potential zoonotic infections in their early stages [105]. Early diagnosis not only enhances patient outcomes but also aids in containing the spread of diseases by enabling prompt isolation and treatment [106]. Delays in the transmission of zoonotic diseases can adversely affect disease prediction in humans. These delays can be influenced by various epidemiological factors, with the most significant factor being the incubation period of the infectious agent in the vector and the incubation period of the infection in the host [31]. AI technology can help address this challenge. For instance, research has been conducted on the use of the Levenberg-Marquardt method to train a backpropagation neural network (LM-BPNN) for intelligent numerical computation [107]. This model was developed specifically to analyze the spread of COVID-19, considering cross-immunity and time delay, and establish a classification model for susceptible, infected, recovered, and cross-immune individuals. By training this model with data, it was possible to reliably predict the incubation period of COVID-19, thereby enhancing the accuracy of disease prediction for this particular virus.

AI provides novel approaches to identify potential therapeutic targets and accelerate the discovery of new drugs. Leveraging techniques, such as virtual screening, AI algorithms can analyze a vast database of chemical compounds and predict their interactions with disease-causing agents [90]. This approach expedites the drug discovery process and holds promise for the development of effective treatments for zoonotic diseases.

Although AI has enabled significant advancements, its applicability in the prediction, diagnosis, and treatment of zoonotic diseases is constrained by certain limitations. One of the primary constraints is the availability and quality of data. AI models rely on large training datasets; however, standardized and comprehensive data on zoonotic diseases are lacking [108]. Factors such as small sample size, underreporting, and biases in the data limit the generalizability of AI models. Additionally, privacy restrictions can pose obstacles to accessing relevant data. As AI in healthcare increasingly utilizes nontraditional data sources, new challenges arise in terms of data curation and ethical considerations [109]. Another limitation is the difficulty AI algorithms face in capturing the complexity of biological systems and disease pathways [110]. These algorithms may fall short in comprehensively elucidating key interdependencies, thereby limiting their capacity to mirror human clinical reasoning. Neglecting to incorporate expert domain knowledge in the design of AI models is another common pitfall, particularly in tasks such as causal diagnosis. Furthermore, several AI tools for zoonotic diseases primarily undergo retrospective testing and lack real-world validation [111]. Their effectiveness in various clinical conditions remains unconfirmed until extensive prospective evaluations are conducted. Even rigorously evaluated models can become outdated as pathogens evolve or as new diagnostic methods emerge. Furthermore, the risks associated with over-reliance on AI recommendations can erode human oversight and accountability [112]. “Black box” algorithms, which lack transparency, exacerbate this problem [113]. The biases present in the training data can propagate silently, leading to a biased output. Additionally, if healthcare users lack a sufficient understanding of AI, they may misuse or misinterpret the outputs of these models. While AI shows promise, it should be seen as a tool to augment clinical expertise and standardize protocols for managing zoonotic diseases rather than as a replacement. By carefully designing and judiciously applying them, we can leverage the strengths of AI while safeguarding it against its weaknesses. Addressing these multifaceted limitations is crucial to drive future studies in this field.

Looking ahead, several key areas warrant further research and development. First, the integration of AI with genomics and proteomics has the potential to provide a deeper understanding of the molecular mechanisms underlying zoonotic diseases. This integration could lead to the identification of more accurate diagnostic markers and targeted therapies. The amalgamation of diverse omics datasets utilizing AI techniques presents significant opportunities for advancing precision medicine and transforming disease surveillance capabilities. For instance, AI techniques that jointly analyze genomic, transcriptomic, proteomic, and metabolomic data from the same biological samples can unveil intricate biomolecular relationships and regulatory mechanisms underlying disease pathogenesis [114]. Researchers have already devised multi-omics fusion algorithms to identify novel diagnostic and prognostic biomarkers as well as molecular subtypes of cancer, resulting in more precise therapies [115]. At the public health level, a combination of surveillance data from genomics, medical records, and environmental monitoring can enhance early warning systems for infectious disease outbreaks [116]. Other studies integrated livestock microbiome data with soil, precipitation, and land-use data to forecast livestock disease risks using ML [117]. Centralized data platforms are emerging to facilitate the integration of dispersed multiomics datasets for large-scale AI model development. For instance, Bessani et al. developed a Hadoop-based platform for secure storage, sharing, and parallel processing of genomic data in the BiobankCloud project. Hadoop is a distributed system architecture that is known for its high fault tolerance. The entire system is open source and supports secure data sharing between different distributed Hadoop clusters. This platform offers predefined workflows for common tasks in biomedical data analysis, such as variant identification and differential transcriptome analysis using RNA-Seq and miRNA-Seq analysis [118]. In April 2020, all 36 university hospitals in Germany established university medical networks using a large-scale data-sharing approach. The CODEX project aims to develop a nationwide COVID-19 data exchange platform to coordinate COVID-19 action plans, diagnostic and treatment strategies, and collaborative research activities [119]. Such extensive biomedical data can act as a catalyst for advanced analytics. Overall, the combination of comprehensive omics data with clinical and environmental data presents immense potential for unraveling disease mechanisms, predicting outbreaks, developing more precise diagnostics and treatments, and ultimately, enhancing healthcare through AI. However, these advancements require extensive data sharing and collaboration among researchers, public health entities, and technological partners.

Additionally, the ethical implications surrounding the use of AI in zoonotic disease control, such as privacy concerns and bias in data analysis, need to be addressed to ensure the responsible and equitable deployment of these technologies [120,121]. As highlighted during the Verena One Health Risk Tech Forum held at Georgetown University's Center for Global Health Science and Security in 2021, AI has a significant potential to revolutionize healthcare (Carlson et al., 2021). However, this raises important concerns regarding the transparency, interpretability, and potential bias of AI systems. As AI algorithms make increasingly complex decisions in areas such as diagnosis and treatment recommendations, it is crucial to ensure that these systems are transparent and interpretable for healthcare professionals [122]. The use of opaque “black box” models can lead to mistrust of AI and hinder its adoption in clinical settings. To address this, steps must be taken to enhance transparency through techniques such as visualizing model logic, embedding explanatory ability directly into model architectures, and generating explanations for individual predictions [123]. Furthermore, AI systems may unintentionally perpetuate or introduce biases based on factors such as gender, ethnicity, socioeconomic status, and geographic location [109,124]. Bias can arise from imperfect training data that fail to adequately represent diverse populations. Mitigating algorithmic bias requires diversifying data sources, auditing algorithms for discrimination, and proactively monitoring model performance across demographic groups [125]. Researchers have proposed techniques such as adversarial debiasing, which employs an adversarial network to remove sensitive attributes from datasets [126]. Healthcare organizations adopting AI must prioritize transparency, interpretability, and fairness [113]. Extensive testing and validation focused on detecting biases is essential before clinical implementation. Rigorous governance frameworks and ethics boards can provide an overview of the societal impact of AI on healthcare [112]. By deliberately building and evaluating ethical AI systems, the healthcare community can harness the benefits of this technology while safeguarding patients. Patient-centered design principles must remain at the core of healthcare AI development and application.

Moreover, effective collaboration and data sharing among researchers, public health agencies, and technology companies are pivotal to fully harness the potential of AI in zoonotic disease research. Scientists worldwide are striving to establish a database based on the “One Health” concept to prevent and manage zoonotic disease outbreaks [127]. Open-access databases, standardized protocols, and interdisciplinary collaborations will facilitate the development of robust AI models and accelerate the translation of research findings into clinical practice. Although AI offers significant potential, there are notable challenges in translating the proof-of-concept into real-world clinical and public health workflows [128]. A key obstacle is the lack of integration with existing health IT systems, which hampers adoption. AI tools that do not consider the needs of clinical end users and fail to seamlessly interoperate with electronic health records, diagnostic equipment, and hospital databases will struggle to impact patient care [128]. Privacy concerns and legal uncertainties surrounding the access of patient data for model development require attention. Another crucial challenge is to demonstrate the efficacy and safety of regulators before allowing their clinical implementation. Most AI models only demonstrate predictive power on retrospective datasets [129]. Rigorous real-world validation through prospective studies and randomized controlled trials is necessary to establish clinical utility [130]. The lack of reproducibility and bias mitigation in current biomedical AI models also leads to skepticism and slow approval. Therefore, gaining support from healthcare professionals is vital. Change management involves demonstrating how AI improves medical decision-making and user experience without causing disruption [131]. Healthcare providers already face burnout and may be skeptical of “black box” recommendations. User-centered design and trust-building are essential for successful adoption. Adequate training of end users on the capabilities and limitations of AI is also crucial to preventing the risk of its misuse and the tendency to excessively rely on it [132]. Finally, the specialized skills required to develop, evaluate, and continually update AI systems pose implementation challenges [133]. Healthcare organizations must foster internal capabilities and establish cross-sector partnerships to sustain AI solutions. The lack of shared standards and best practices further complicates technology replication and scales the costs. Overcoming these challenges requires concerted efforts across policies, regulations, organizational processes, and team capabilities. However, thoughtfully implemented AI has the potential to transform decision-making support in the context of managing infectious diseases.

## 6 Conclusions

Innovative applications of AI in zoonotic diseases show tremendous promise for disease prediction, early diagnosis, and drug development. Harnessing the power of AI can improve our ability to prevent, diagnose, and treat zoonotic diseases, ultimately safeguarding public health and mitigating the global impact of these infectious threats.

## Compliance with ethical standards

This research involved no experimental animal or human participant.

## Funding

This research did not receive any specific grant from funding agencies in the public, commercial, or not-for-profit sectors.

## Data availability

Data sharing is not applicable to this article because no datasets were generated or analyzed during the current study.

## Author contributions

Conceptualization, methodology, and writing—original draft preparation: W.G. and C.L.; writing—review and editing: W.G., C.L., M.G., Q.Z., X.Y., and L.Z.; supervision: L.Z.; funding acquisition: L.Z. All authors have read and agreed to the published version of the manuscript.

## Declaration of competing interest

The authors declare that they have no known competing financial interests or personal relationships that could have appeared to influence the work reported in this paper.
